# A data mining approach in home healthcare: outcomes and service use

**DOI:** 10.1186/1472-6963-6-18

**Published:** 2006-02-24

**Authors:** Elizabeth A Madigan, Olivier Louis Curet

**Affiliations:** 1 Both of the Frances Payne Bolton School of Nursing, Case Western Reserve University, USA

## Abstract

**Background:**

The purpose of this research is to understand the performance of home healthcare practice in the US. The relationships between home healthcare patient factors and agency characteristics are not well understood. In particular, discharge destination and length of stay have not been studied using a data mining approach which may provide insights not obtained through traditional statistical analyses.

**Methods:**

The data were obtained from the 2000 National Home and Hospice Care Survey data for three specific conditions (chronic obstructive pulmonary disease, heart failure and hip replacement), representing nearly 580 patients from across the US. The data mining approach used was CART (Classification and Regression Trees). Our aim was twofold: 1) determining the drivers of home healthcare service outcomes (discharge destination and length of stay) and 2) examining the applicability of induction through data mining to home healthcare data.

**Results:**

Patient age (85 and older) was a driving force in discharge destination and length of stay for all three conditions. There were also impacts from the type of agency, type of payment, and ethnicity.

**Conclusion:**

Patients over 85 years of age experience differential outcomes depending on the condition. There are also differential effects related to agency type by condition although length of stay was generally lower for hospital-based agencies. The CART procedure was sufficiently accurate in correctly classifying patients in all three conditions which suggests continuing utility in home health care.

## Background

Home healthcare is the provision of patient care delivered in the home. Although many older Americans receive assistance in their homes, home healthcare has been differentiated by the type of provider where healthcare professionals deliver home healthcare while paraprofessionals (primarily aides and homemakers) provide home care [1]. In the US in the 1990s, there was a paradigm shift in the financing of home healthcare services by Medicare. A significant decline in the use of home healthcare services took place after the reforms of the Balanced Budget Act (BBA) of 1997. As a consequence of these changes, both the quality and quantity of home healthcare services have been impacted [2-5]. After the change in Medicare policy, the focus of the US home healthcare industry shifted from various medical and nursing models (accompanied by many protocols for data collection) to more standardized models (in which some clinical data are collected in a systematic manner).

### Home healthcare

Home healthcare is an important area of study for several reasons: 1) many people use home healthcare yet there is still a lack of understanding of the effectiveness of home healthcare; 2) there is a lack of evidence explaining discrepancies in patient outcomes; 3) to date there has been little combining of advanced statistical and/or probabilistic analyses to this domain area; and 4) some recent studies have shown that there have been many failures in home healthcare industry and that learning from those failures may help us ensure that patient safety can be improved. One such study [6] found that failures came mainly from either low support from home health agencies or insufficient coordination with patients. Finally, there is additional pressure for home healthcare agencies to provide high quality care as information on patient outcomes is published and directly accessible from the Internet, known as Home Health Compare . This availability of ready-made outcome information may help in improving home healthcare services in the US [7], although the extent of consumer use has yet to be determined.

However, it should be borne in mind that assessing patients' outcomes across home healthcare providers is a difficult task. One of the reasons for the level of difficulty of such a task is the multiplicity of all possible risk factors and patient outcomes, as well as their levels of variability and inter-dependence or interrelationships. Furthermore, many types of patient level characteristics (such as diseases, health histories, environment circumstances, cognitive abilities, physical resources, and so on) are often very distinct. For these reasons, it has been suggested that traditional statistical techniques are not well suited for analyzing drivers of health outcomes and no traditional approach can control all external factors affecting patient health outcomes [8]. There is also an increased interest in how information techniques can help model and monitor patient health outcomes to improve the quality of care. For example, it has been shown that the findings from computer-driven analyses of patient health outcomes can lead to quality improvement [9]. Another example of such research analyses has shown that home healthcare agencies which direct their efforts on key risk factors (such as for specific clinical, functional and demographic factors) can help understand and therefore decrease the rate of patients' re-hospitalizations [10]. Other past research also has illustrated the need for analyzing patient outcomes for those with heart failure [11] where interventions have been only moderately successful.

Standard statistical techniques have shown their limitations and authors have turned their attention to the application of data mining to home healthcare. Data mining can be defined as searching for meanings among patterns of datasets that can be generalized to new datasets. In a recent published paper, data mining has been applied to home healthcare research [12] where it was found that this technique can help enhance safety of patients in home healthcare settings. The use of data mining has also been established in reducing rates of medication errors[13].

### CART as the approach selected for this research

There are many data mining approaches. Approaches include clustering techniques (i.e. genetic algorithms, regression models, decision trees, k-nearest neighbor, neural networks, and rule induction). Some of these data mining approaches can be used for predictive modelling, including tools such as ID3, C4.5 [14], C5, CART [15], and CHAID [16]. CART (Classification and Regression Trees) is a non-parametric approach which has been used in many research domains, including medicine, business, and applied sciences. Past research in healthcare includes the use of CART as a technique to recognize critical situations derived from specific laboratory results [17], identification of nursing diagnoses [18], or in quality and safety issues of healthcare delivery [19]. CART has also been used (as part of a case-based reasoning approach) to support a safety culture assessment in order to improve patient safety in healthcare organizations' environments[20].

CART can be used for two main types of classification methods: 1) Prediction (which means the ability to predict any class of previously learnt or unlearnt data) and 2) Interpretability (which relates to the level of understanding and insight provided by the model). CART has also been recognized as one of the most powerful approaches to design, test and use advanced, mathematically-based decision trees. Since CART uses trees to represent models, it is intuitively more appealing than other model-based classifiers such as logistic regression.

CART has many advantages. It is non-parametric and fast. It is also easy to interpret the tree-based findings. There is no need to have an exact distribution among predictor variables, many types of data can be analyzed (i.e. Boolean, symbolic, textual, numeric) and CART performs well with missing values. CART designs trees by using binary splits among data and it makes use of all available variables while remaining stable to the outliers skewness.

## Method

### Explanation of CART

CART-based decision trees are created using three steps. First, each node is split in a tree: the problem is how to select "children nodes" in such a way that these "children nodes" are "purer" than the "parent nodes." Second, the analyst prunes the branches and nodes – otherwise the trees can be too large for meaningful analysis. The pruning can be done either by stopping the splitting process once some "goodness of split" criteria have failed to be met or when the tree is complete (i.e. when all data has been clustered), nodes and branches need to be pruned. Third, predicted values can be assigned to terminal nodes. For example, if the group of patient profiles (i.e. successful outcomes) has the greatest representation, then this outcome "successful" value will be assigned to the node.

CART-derived trees include a root node, parent nodes, and children nodes. The splits derived from CART help by dividing the "parent nodes" of the trees into two "children nodes." The CART algorithm splits parent nodes by analyzing whether specific constraints have been met or not at each decision node. The aim of the CART algorithm is to find the splits which create homogenous children nodes. The tree expands until the nodes develop into more and more homogenous nodes.

The rules which split among data are derived from different *impurity functions *(or diversity functions). There are different types of impurity functions used, including entropy (which purpose is to select attributes minimizing impurity) and Gini index (which relates to the measure of impurity). Both the entropy and the Gini approaches deal appropriately with data diversity at any given node. From the application of these rules it is possible to use a splitting rule to separate a node into two further nodes by calculating impurity loss. This is called the "goodness of the split" which is defined primarily as the decrease in impurity.

Given:

*t *a particular node; *s *a particular split for that node

*P*_*R *_= proportion of cases at node *t *that go into the right child node *t*_*R*_

*P*_*L *_= proportion of cases at node *t *that go into the left child node *t*_*L*_

and given for impurities :

*i(t*_*R*_*)*= impurity of the child node on the right

*i(t*_*L*_*) *= impurity of the child node on the left

The Gini impurity measure for node t is defined as follows [15]:

Δ*i(s, t) = i(t) -P*_*L*_*i(t*_*L*_*)-P*_*R*_*i(t*_*R*_*)*

**Figure 1:**
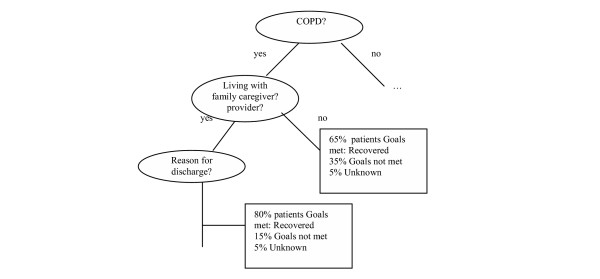
Example of part of a cluster tree.

The Gini impurity maximizes average purity of the two children nodes. Splitting criteria are derived from the different purity measures among children nodes. The selected splits are those that decrease the Gini index most. CART keeps splitting data until all the terminal children (or nodes) have very few data or are all the data under the nodes are similar (or "pure"). This is usually the stage when most CART approaches stop growing trees. Using this inductive method it is possible to find possible interactions among independent variables. The construction of a CART tree is done by applying splitting rules to data sets. The example shown in Figure 1 gives an illustration for outcome of home healthcare of COPD patients. The partial tree indicates that 65% of patients with COPD recovered when they have not been living with their family caregiver, but 80% with the same condition recovered when they were living with their caregiver. The result of such a process allows the user to understand and explore further the most significant variables of the problem domain which have most information-richness so that the shortest tree possible can be created.

### Data

Data were obtained from the 2000 National Home and Hospice Care Survey (NHHCS) which is an ongoing survey conducted by the National Center for Health Statistics (NCHS) of the CDC. The NHHCS is performed periodically, generally every two years. A stratified random sample of agencies is selected within some specific major metropolitan areas. For each agency, data collection staff working for the NCHS visits the agency and collects data on the agency itself, current patients, and discharged patients. Current patients are selected from a random sample of patients on the agency roster while discharged patients are randomly selected from one month's discharges. Data collectors are directed to sample six current and six discharged patients and a standard set of data elements are derived from the patient chart or conversations with the agency staff [21].

Activities of daily living (ADLs) and instrumental activities of daily living (IADLs) were obtained for both current and discharged patients by querying whether the patient received help from the agency in bathing or showering, dressing, easting, transferring, walking, using the toilet room (ADLs), doing light housework, managing money, shopping for groceries or clothes, using the telephone, preparing meals, or taking medications (IADLs). The possible answers were yes (received agency help), no (did not receive help), don't know, and not applicable [21].

The data were cleaned and prepared for the CART analysis by removing specific fields that were not part of the problem domain but which were still included in the data set (for example, marital status). Those variables which had too many missing values were also deleted from the sample because of the exploratory nature of the data mining. After the cleaning phase was completed, a total of 580 records were kept for the three identified conditions (chronic obstructive pulmonary disease or COPD, hip replacement, and heart failure) with respective sample sizes n = 206, 68, and 306. Although the hip replacement sample was substantially smaller than the other two diagnostic groups, it was retained because it represented a surgical population who may have had different discharge and length of stay outcomes than the more medically (i. e. chronically ill) population of those with heart failure and COPD.

The different variables used for CART processing were taken from NCHS files. These were selected based on past empirical studies identifying the variables most commonly found to be associated with either use of home healthcare services or patient outcome profiles.

### Data set problems

Dealing with the home healthcare data posed specific problems. First, the data provided by the CDC comes in large samples. Tens of thousands of patient profiles exist. For this study some specific choices were made regarding the types of conditions to be used in order to narrow down the sample size. For this study it was decided for example to focus on three conditions only: COPD, hip replacement, and heart failure. These three groups represent high volume medical and surgical conditions in home healthcare. For each of these samples we obtained a sample size large enough to work with. Second was the confidential nature of information. While the information was relatively easy to obtain (FTP via the National Center for Health Statistics web site), there is no patient level identifying information beyond the survey. One of the direct consequences is that it is impossible for research purposes to follow up on patients' status (for example a patient case might be classified as having a "successful outcome" but two days later has to be returned to a hospital or nursing home). Third, the presence of missing data: as for many other medical and nursing domains, the data sets have missing values for certain attributes. There are many reasons for this. For example, the complete medical records were not available when the survey was being performed or the agency did not provide a specific type of service. Fourth, there were variable specific issues. For example, the variable "length of stay" reports the number of days on service which varies widely and thus has a wide standard deviation. This is a common issue with utilization data [22]. One question tested as part of the research was whether or not it made sense to normalize the data by using log transformations. After testing, it was decided not to normalize it.

## Results

The first selected outcome value was "Reasons for discharge". Reasons for discharge were reclassified to:

1 died (original classification: 11);

2 transferred to nursing home, SNF, hospice (original classification: 07, 08, 09);

3 hospitalized (original classification: 06);

4 discharged – goals met (original classification: 01, 02, 03);

5 services no longer needed (original classification: 04);

6 other (original classification: 05)

Both "discharged-goals met" and "services no longer needed" were considered positive outcomes. The wording for services no longer needed on the discharged patient survey was "services no longer needed, treatment plan completed" which differentiates patients whose conditions have improved (discharged-goals met) from those for whom services were not needed because the treatment was complete, even though their condition may not have substantially improved. For example, patients who have a chronic condition and have had an acute exacerbation may have a stabilized condition but not really "improved." The variables used for the three conditions (COPD, heart failure, hip replacement) include 1) age (reclassified as <85, and >= 85), gender, ethnicity; 2) whether caregiver is living at home with patient or not; 3) ADL and IADL activities for which the patient receives agency assistance; 4) type of agency, including type of ownership of agency; and 5) source of payment.

### COPD (n = 206)

For patients with COPD, the findings from CART indicate that age at admission is the key discriminating variable influencing reasons for being discharged. The two main groups of patients are those who are less than 85 years, and those who are 85 or older. For those who are less than 85 years (n = 176), the next differentiating factor was ethnicity. For whites less than 85 years old (n = 168 patients), 72 died (42.8%), 12 were transferred to nursing home, SNF, or hospice (7.1%); 17 were hospitalized (10.1%); 38 discharged with goals met (22.6%); 24 had services no longer needed (14.4%); 5 were classified as "other" (3.0%). The results are shown in Table 1. For those less than 85 years old with other ethnicities (blacks, Asian, Hispanic, American Indian or Alaskan native), there were only 8 patients, 3 of them were discharged with goals met.

For those patients who are older than 85 years (n = 30), the next differentiating factors were type of agency (i.e. proprietary vs. non profit and others), gender, and caregiver relationships. For this set, it seems that better outcomes (i.e. patients discharged when goals met) are associated with three factors 1) when type of agency is "non profit and others" (as compared with proprietary), 2) when caregiver is a spouse, parent or child and those whose caregivers are other relatives or acquaintances (i.e. brothers, sisters, neighbours, friends, etc.) and 3) gender (for COPD, men had better outcomes than women).

### Heart failure (n = 298)

CART results from the sample also show interesting findings. The major differentiators were age at discharge, and whether the agency was operated by a hospital or not with age having the most significant effect. Specifically, patients under age 85 years were more likely to be hospitalized (14 to 16%) versus less than 10% for those age 85 and older. Being discharged with goals met was more frequent in those under 85 and those over 85 whose agency was hospital operated (30%, 29%, 24%, respectively) versus 14% of those 85 and older whose agency was not hospital operated.

For this sample, 8 patient profiles (2.6%) were not taken into consideration because their primary source of payment has not been determined (i.e. payment source classified as "other", "not yet determined", or the answers was noted as "not applicable"). Therefore, the new sample size under study is n = 298. Table 3 shows the results.

### Hip fracture (n = 66)

Primary source of payment, gender, and age at discharge are the main variables explaining reasons for discharge. Two patient profiles were excluded from the sample because these profiles skewed the results too significantly. For the rest of the sample (n = 66), age at discharge was the main predictive factor linked with successful outcome. Forty patients out of 66 (or 60.6%) were less than 85 years old, and 26 (or 39.4%) were older. The findings for the younger group (as shown in Table 4) indicate that more than 80% of younger patients having hip surgery having Medicare, Medicaid, private insurance or own income were discharged with goals met or had services no longer needed. However, for the 26 patients older than 85 years old, results show that about two thirds of older patients were discharged with goals met or had services no longer needed.

### Length of stay

Length of stay was selected as an outcome as it represents a utilization outcome. In this time period (2000) during the change to the prospective payment system, there is less published data regarding factors associated with utilization of home healthcare services.

### COPD (n = 203)

For this analysis, three patients were excluded because their profiles seriously skewed the data. These patients are the only Hispanic, American Indian or Alaskan Natives with length of stay varying from 4 to 1,424 days. These three patients were excluded for two main reasons 1) there were too few with this minority status to analyze and 2) the variation in their length of stay skewed the results.

For the rest of the dataset, age of discharge is the main differentiating factor. For those patients whose age is less than 85 years, (n = 169), the type of agency (i.e. proprietary vs. non profit and others) and the primary source of payment are closely linked to length of stay. CART findings show that for patients who are less than 85 and when the agency is proprietary (n = 61), then the length of service (in days) is 95 days (SD = 187 days). For those patients with age at discharge 85 years or more, (n = 34), the average length of stay was 142 days (SD = 239 days).

### Heart failure

There are two major discriminating variables influencing length of stay among patients with heart failure. They are 1) primary source of payment and 2) whether the agency is operated by a hospital or not. For patients with Medicare whose agency is being run by a hospital (n = 91), the average length of service is 49 days (SD = 60 days). For patients with Medicare, and for whom agency is not run by a hospital (n = 168), the average length of service is 77 days (SD = 173 days). However, for those who have other funding than Medicare (i.e. Medicaid, other governmental assistance, or other), the average length of service is 202 days (standard deviation = 333 days). Thus, non-Medicare patients received services for substantially longer periods.

### Hip fracture (n = 68)

CART findings indicate the main parameter driving length of stay is whether the agency is operated by a hospital or not. When agency is operated by a hospital, the average stay is 37 days (SD = 29). When agency is not operated by a hospital, the average stay is 78 days (SD = 118 days).

### Performance analysis

As part of this research it was decided to compute classification and misclassification rates. Classification can be defined as the relative number of unlearnt records which are accurately assigned to a cluster. When assessing the classification / misclassification accuracy of CART, many ways can be used. Some ways of testing accuracy results can be done by calculating the overall sum of the total number of correct predictions under a "right" node and a correct prediction of the "wrong" node category. The sum of "right" or "wrong" results is then divided by the total number of cases. Average classification scores as well as their standard deviation are then calculated. A perfect classifying model should have a 100% level of accuracy for the "correct" nodes and the "wrong" nodes.

As part of this approach, a method derived from past research [23] was used. Curet's approach involved classification accuracy being calculated by analyzing the consistency of results (e.g. predicted outcome label in final nodes of the tree as compared to given outcome label), using different sizes of training data, and analyzing the standard deviation of "hits" (when all the predicted outcome label match the given outcome label), "partial hits" (when at least one the predicted outcome label match the given outcome label) and "miss" (when none of the predicted outcome label matches the given outcome label).

The data for each sample in this research was portioned randomly into training sets (33%, 66% and 95%).

### COPD

**Figure 2:**
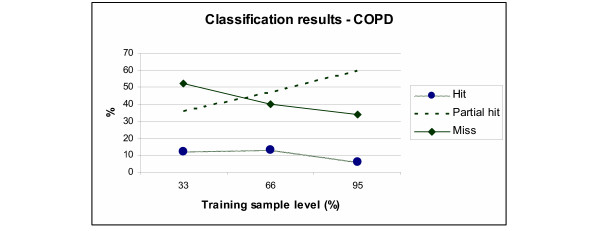
Breakdown of classification results for COPD patients with three levels of training samples.

With training set of 33% of sample data (i.e. 68 patients out of 206) CART predicted 51% of outcomes correctly ("hits" and "partial hits"). The results improved further with a larger training set (e.g. 66%, n = 136): in that case, CART found a perfect hit more than 11% of the time, it also identified similar outcomes (partial hits) for 59% of the sample (see Figure 2). It seems that the "perfect hits" levels for this sample are fairly insensitive to increased sizes of training samples, and the partial hits are the major beneficiaries of the decrease in the level of missed outcomes. The results increased marginally only with a training set of 95% (n = 196): about 35% of sample on average were still missed.

### Heart failure

**Figure 4:**
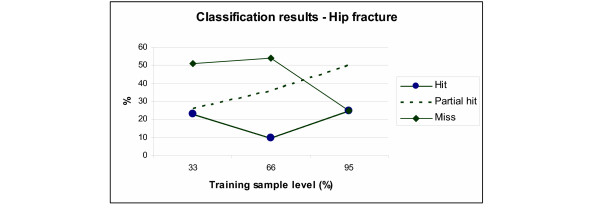
Breakdown of classification results for patients with hip fracture, with three levels of training samples.

CART was able to identify correct outcomes ("hits" and "partial hits") in about 71% of sample using a training set of 33% (N = 100) (Figure 4). The results were improved marginally with a larger training set of 66% (N = 201), and the main improvement at this level is seen on direct "hits" only (an improvement of about 60%) rather than "partial hits". Here again, using a training set of 95% (N = 289), the improvements were marginal.

### Hip fracture

Here the results were interesting too (as shown in Figure 4). The results of the "partial hits" with the three levels of training (33, 66, and 95%, N = 22, 45, 65) were respectively 24, 37 and 50% (an improvement of more than 100%). Interestingly also, the percentage of "missed outcome" went from 51% to 27%, a significant improvement.

These classification results can be used to support explanatory models to distinguish among different variables, outcome values and outcome records. These classification results can be also be used to support extrapolative models to predict the class labels of new patient records. This shows that when applied to home healthcare data, CART can help classify patient profiles in a reliable way. Cluster trees are easy to understand although some authors prefer rule-based representation (in the form of IF-THEN-ELSE), but many agree that the difference between visualization of clusters, groups, and rule based formalisms is mainly philosophical. More importantly, the results from CART analysis offer an interesting explanation of those factors that may influence patient outcomes.

## Discussion

Approaches like the one used in this paper have utility for policy, practice and future research. For example, data mining can be used to support a benchmarking approach in order to compare home healthcare agencies. Benchmarking initiatives have been mentioned in the literature; for example in New Jersey where a benchmarking project examined the Outcome and Assessment Information Set (OASIS) and Outcome Based Quality Improvement (OBQI) reports published by home healthcare agencies [24]. In some ways, the publicly reported home healthcare outcomes are benchmarks in that national results are presented for consumers and others to use to compare a specific agency's performance with national results, although benchmark infers that a "correct" or "ideal" rate has been established, which is not the case. Thus, the use of a data mining approach could identify the dependencies and interactions that influence outcomes so that risk adjustment methodologies can be improved in accuracy and actual benchmarks could be established.

Of note, the persistent age related differences found in the present study suggest that any risk adjustment methodologies stratify or adjust outcomes using age 85 reflecting that this group of oldest old have more positive outcomes for some kinds of measures (e. g. lower hospitalization rate for patients with COPD) and less positive outcomes for other kinds. The findings from the present study indicate the complexity of outcome evaluation in a heterogeneous population, some of whom may be of advanced age but in otherwise good health and functionally very able.

Data mining approaches could also be used to establish and validate guidelines for clinical practice for home healthcare, which have been lacking [25]. Next steps could be to include larger sample sets of data, or testing those finding on other medical conditions. Other future research could investigate further differences between US regions or according to patient socioeconomic class, for example rural and/or low income patients.

Problems with CART still include the fact that a cluster tree does not always show the "best" picture of a problem domain – a specific node or class might hide another, as overtraining may occur. Another potential issue with CART is that the trees are not always perfectly stable: for example, a new variable can change the whole branches and nodes. A derived consequence is that although trees are optimal at each node, the optimality does not apply to the overall tree. Taking these problems in consideration, other statistical techniques could be used in combination of CART to try to improve on these results. For example, the bagging technique (a technique to reduce variance) might be considered as this technique calculates a "model" tree averaging the number of trees derived from boot-strapped samples included in the original training set of data. Another technique to improve results might be the use of boosting which grows the tree several times, but each time the trees are grown again, the data (i.e. patient records) which was misclassified are assigned a greater weight in order to improve the predictive nature of the tree. Another future research area could be to improve validation results by analyzing optimum size of trees for better results for example.

There are a number of cautions with the findings from this study – first we do not imply cause and effect relationships with CART, we are simply identifying the relationships of the data. For example, "type of agency" is associated with some differences in outcomes yet we cannot conclude that agencies of specific types are "superior" to other types. Perhaps the most important caution is that we do not have the comorbid or multimorbid conditions for the patients in the sample. This would, in part, explain some of the findings that may be counter-intuitive. For example, older patients are generally found to achieve less positive outcomes following hip fracture, yet our findings suggest otherwise. We suspect there is one primary reason for this – many patients over 85 who have hip surgery go to a skilled facility of some type for rehabilitative services following hospitalization. Thus, the patient group who received home healthcare were likely to have better functional and health status at hospital discharge and home healthcare agency admission. Another limitation is the structure of the questions related to functional status, which ask whether the patient requires agency assistance with the particular ADL and IADL items rather than the patient's actual performance of the item. This limits the ability to judge actual performance as compared to the amount of assistance provided by the agency and also examine differences by functional status ability. Finally, the sample sizes were relatively small, particularly for hip fracture and, thus, the results need to be interpreted cautiously.

The data used for this data mining experiment was prior to the major changes in Medicare reimbursement brought about by the Balanced Budget Amendment of 1997. At present, agencies function under a prospective payment system, which began in 2001, where there is a base rate of pay which is adjusted up or down based on specific patient characteristics in three domains: functional, clinical and service utilization. During the year 2000, agencies were operating under an interim payment system which was, in some ways, more severe because it was based on historical payment instead of risk adjustment. Thus these data reflect this interim payment system time frame. More recent data have not been released by the NCHS from subsequent home healthcare surveys. The same findings here may or may not hold in the PPS era.

The oldest old, those 85 and older, are receiving increasing attention in the gerontological sciences because of the growth of this segment of the older population. Within home health care, these oldest old people are also growing in number and, based on these findings, experience differential outcomes compared to those less than 85. Additional research in this area might also examine more closely the oldest old patients in home healthcare to determine if, at the patient level, they experience differential outcomes compared to the younger old and whether there are differences in the validity of the risk adjustment models currently in use.

## Competing interests

The author(s) declare that they have no competing interests.

## Authors' contributions

EAM suggested the idea and both authors explored the possibility of using the data set.

OLC performed the CART analyses. The CART sections were written by OLC. Collaborative writing by both was done for the background and discussion/conclusions sections.

## Pre-publication history

The pre-publication history for this paper can be accessed here:



## Figures and Tables

**Figure 3:**
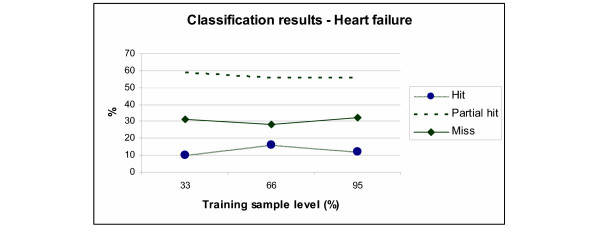
Breakdown of classification results for heart failure patients with three levels of training samples.

**Table 1:** Differentiators – reasons for discharge for COPD patients

	Age at admission <85 AND ethnicity = white (n = 168)	Age at admission >85 AND ethnicity = white (n = 29)
Died	72 (42.8%)	19 (65.5%)
Transfer	12 (7.1%)	0
Hospitalized	17 (10.1%)	1 (3.4%)
Discharged with goals met	38 (22.6%)	2 (6.8%)
Services no longer needed	24 (14.4%)	4 (13.8%)
Other	5 (3%)	3 (10.3%)

**Table 2:** Differentiators – reasons for discharge for COPD patients

	Age discharge <85 AND agency operated by hospital (n = 61)	Age discharge <85 AND agency non for profit or others (n = 108)	Age of admission and age discharge > 85 AND any type of agency (n = 30)
Died	22 (36%)	50 (46.3%)	19 (63.3%)
Transfer	8 (13.1%)	3 (2.8%)	1 (3.4%)
Hospitalized	7 (11.4%)	9 (8.3%)	2 (6.7%)
Discharged with goals met	13 (21.3%)	27 (25%)	4 (13.3%)
Services no longer needed	9 (14.9%)	15 (13.9%)	4 (13.3%)
Other	2 (3.3%)	4 (3.7%)	0

**Table 3:** Differentiators – reasons for discharge for patients with heart failure

	Age discharge <85 AND agency operated by hospital (n = 64)	Age discharge <85 AND agency not operated by hospital (n = 119)	Age discharge = 85 AND agency operated by hospital (n = 37)	Age discharge = 85 AND agency not operated by hospital (n = 78)
Died	13 (20%)	39 (33%)	16 (43%)	48 (62%)
Transfer	3 (5%)	8 (7%)	0 (0%)	4 (5%)
Hospitalized	10 (16%)	17 (14%)	2 (5%)	6 (8%)
Discharged with goals met	19 (30%)	34 (29%)	9 (24%)	11 (14%)
Services no longer needed	18 (28%)	21 (18%)	8 (22%)	6 (8%)
Other	1 (2%)	0 (0%)	2 (5%)	3 (4%)

**Table 4:** Differentiators – reasons for discharge for patients with hip fracture

	Age <85 (n = 40)	Age >85 (n = 26)
Died	1 (2.5%)	1 (3.8%)
Transfer	3 (7.5%)	3 (11.5%)
Hospitalized	3 (7.5%)	4 (15.3%)
Discharged with goals met	12 (30%)	11 (42.3%)
Services no longer needed	20 (50%)	7 (26.9%)
Other	1 (2.5%)	0
